# Patient-reported outcomes as early warning signs of flare following drug cessation in rheumatoid arthritis

**DOI:** 10.1136/rmdopen-2025-005442

**Published:** 2025-04-01

**Authors:** Leher Gumber, Fiona Rayner, Theophile Bigirumurame, Bernard Dyke, Andrew Melville, Sean Kerrigan, Andrew McGucken, Najib Naamane, Jonathan Prichard, Christopher D Buckley, Andrew Filer, Iain B McInnes, Karim Raza, Stefan Siebert, James MS Wason, Wan-Fai Ng, Amy E Anderson, John D Isaacs, Kenneth F Baker, Arthur G Pratt

**Affiliations:** 1 Translational and Clinical Research Institute, Newcastle University, Newcastle upon Tyne, UK; 2 Musculoskeletal Unit, Newcastle upon Tyne Hospitals NHS Foundation Trust, Newcastle upon Tyne, UK; 3 Population Health Sciences Institute, Newcastle University, Newcastle upon Tyne, UK; 4 NIHR Birmingham Biomedical Research Centre, University Hospitals Birmingham NHS Foundation Trust, Birmingham, UK; 5 School of Infection and Immunity, University of Glasgow, Glasgow, UK; 6 Newcastle Clinical Trials Unit, Newcastle University, Newcastle upon Tyne, UK; 7 Kennedy Institue for Rheumatology, University of Oxford, Oxford, UK; 8 Department of Rheumatology, Bronglais General Hospital, Aberystwyth, UK; 9 Department of Rheumatology, Sandwell and West Birmingham NHS Trust, Birmingham, UK; 10 University College Cork, Cork, Republic of Ireland

**Keywords:** Rheumatoid Arthritis, Patient Reported Outcome Measures, Autoimmune Diseases

## Abstract

**Objectives:**

Drug withdrawal in rheumatoid arthritis (RA) in remission can reduce toxicity, but with the risk of flare which requires close monitoring. We explored the potential of patient-reported outcomes (PROs) for flare detection among RA patients in sustained remission after conventional synthetic disease-modifying antirheumatic drug (csDMARD) cessation.

**Methods:**

Four PROs (Factors that Limit sustAined Remission in rhEumatoid arthritis (FLARE-RA), EuroQol-5 Dimensions (EQ5D), Routine Assessment of Patient Index Data-3 (RAPID-3) and RA Flare Questionnaire (RA-FQ)) were captured at baseline and at sequential visits until time-of-flare or end of 6-month follow-up as part of the BIO-FLARE prospective cohort study. Flare was defined as any of (i) Disease Activity Score 28 (DAS28)-C reactive protein (CRP) ≥3.2 at any visit, (ii) DAS28-CRP≥2.4 on two visits within 2 weeks or (iii) resuming DMARD and/or steroid therapy despite DAS28-CRP<2.4. Cox regression models with time-varying covariates were fitted to evaluate associations between PRO changes and likelihood of flare. Receiver-operating characteristic (ROC) curves enabled discriminatory changes in each PRO to be compared as a means of identifying flare.

**Results:**

58/121 (47.9%) participants (70.1% females, mean age 64.8 years) experienced a flare. A 1-point change in each PRO score was strongly associated with flare development in the multivariate Cox regression model (p<0.001 in each case). ROC curve analysis confirmed that monitoring adverse changes in PROs from baseline offered robust discriminatory utility for identifying flare occurrence. This was most evident for RA-FQ and FLARE-RA (both areas under the curves 0.90, 95% CI 0.84 to 0.96; p=0.001); for example, an RA-FQ increment of ≥5.5 from baseline identified objective flare with positive and negative predictive values of 80% and 91%, respectively.

**Conclusions:**

Our data support the potential value of remote PRO monitoring of RA patients in drug-free remission to identify flare occurrence.

**WHAT IS ALREADY KNOWN ON THIS TOPIC**

**WHAT THIS STUDY ADDS**

- This is the first study to prospectively evaluate an array of PROs in RA patients following conventional synthetic disease-modifying anti-rheumatic drug cessation.
- When compared with baseline, adverse PRO changes over time according to defined thresholds offer robust discriminatory utility for prompt flare identification; this is particularly evident for RA-Flare Questionnaire and Factors that Limit sustAined Remission in rhEumatoid arthritis-RA.

**HOW THIS STUDY MIGHT AFFECT RESEARCH, PRACTICE OR POLICY**

- Our study supports the use of PRO monitoring for prompt flare identification in the context of drug withdrawal; future studies should evaluate its value in remote settings.

## Introduction

 Rheumatoid arthritis (RA) is a chronic immune-mediated inflammatory disease that can result in joint destruction and permanent disability.^1^ ‘Treat-to-target’ management in the era of targeted therapies has transformed clinical outcomes,^2^ substantially reducing prevalent disease activity.^3 4^ Nonetheless, the unpredictable, relapsing-remitting characteristic of the disease remains a barrier to achieving sustained remission and is a significant impediment to quality of life for many. Indeed, 15%–30% of patients are still expected to experience a disease activity ‘flare’ in any given year, with associated adverse outcomes.^5 6^ The ability to predict and prevent flare among RA patients who have achieved remission therefore remains a major unmet need, with progress in part hampered by challenges in defining flare itself.^7^ Importantly, in this regard, distinguishing flare from daily symptom variation may be defined differently by patients and healthcare providers.^8 9^ Relatively ‘subjective’ symptoms resonant of RA activity for patients, such as impairment of function or well-being, could prove at least as sensitive as ‘objective’ flare definitions more readily recognised by clinicians such as above-threshold increments in the Disease Activity Score (28 joints; DAS28).^10^ Capturing longitudinal variation in patient-reported outcomes (PROs) before and during disease activity fluctuation in people otherwise in clinical remission could help test such hypotheses, prioritising strategies for prompt intervention in case of incident RA flare and even informing efforts to predict and thereby intercept flares.

A number of existing PROs are worthy of consideration for such purposes in the context of RA. Functional status, quality of life measures and fatigue have, for example, each previously been associated with an increased likelihood of RA flare^6^; respective validated tools to measure them include the Health Assessment Questionnaire Disability Index (HAQ-DI),^11^ EuroQol-5 Dimensions-3 Level (EQ5D-3L)^12^ and Multidimensional Fatigue Inventory (MFI)^13^ constructs. The Routine Assessment of Patient Index Data-3 (RAPID-3) tool is an example of a validated PRO for which longitudinal variations reflect those of more conventional measures.^14 15^ Tools designed specifically for detecting RA flare have been developed more recently; namely the RA Flare Questionnaire (RA-FQ) and Factors that Limit sustAined Remission in rhEumatoid arthritis (FLARE-RA) constructs—each seeking to incorporate variables of more relevance for patients as well as objective measures, though yet to be evaluated in the context of disease remission. The notion that such tools might more sensitively detect flare among RA patients previously in clinical remission raises the intriguing prospect that their use for remote monitoring might not only circumvent the need for frequent hospital visits, but also pre-empt flare such that mitigating interventions could be deployed in a timely manner to prevent it.

In this study, we explored the potential of PROs for flare detection in a cohort of RA patients who had been in sustained remission for >6 months at the start of a conventional synthetic disease-modifying anti-rheumatic drugs (csDMARD) discontinuation trial.

## Methods

### The BIO-FLARE cohort and flare definition

The BIOlogical Factors that Limit sustained REmission (BIO-FLARE) study was a multicentre, prospective, single-arm, open-label experimental medicine study of complete csDMARD cessation in patients with RA who had achieved >6 months’ sustained remission on methotrexate, sulfasalazine or hydroxychloroquine as monotherapy or in combination (patients on biological or targeted synthetic DMARDs were excluded).^16^ Briefly, 121 participants who fulfilled the eligibility criteria stopped all csDMARDs. Participants were followed up over a period of 24 weeks from csDMARD cessation or until confirmed flare, whichever occurred first. Flare was predefined as the occurrence of any of the following: (i) DAS28-C reactive protein (CRP) ≥3.2 at any visit, (ii) DAS28-CRP≥2.4 on two occasions within a 2-week period or (iii) clinical indication for steroid rescue therapy and/or DMARD restart despite DAS28-CRP<2.4. Further details of the study design, recruitment and data collection have been previously described.^16^

### Patient-reported outcomes

Participants completed PRO questionnaires at all predefined clinical visits: 0, 2, 5, 8, 12 and 24 weeks after cessation of DMARDs or until point-of-flare, whichever was earlier. Ad hoc PROs were also completed if participants missed a clinical visit or had a flare between visits (online supplemental table S1). Participants could not routinely access joint counts being recorded during study visits, and DAS28 calculations were completed after visit completion. A total of six different self-reported PROs were used as follows.

The HAQ-DI measures function and encompasses 20 questions which span eight categories.^11^ The MFI is a measure of fatigue and comprises five subscales. Each subscale includes four items with 5-point Likert scales.^13^ HAQ-DI and MFI were completed only at baseline and end of study period. The remaining PROs were administered at baseline and at each subsequent clinical visit. EQ5D is a generic measure of health status and consists of five measures: mobility, self-care, usual activities, pain and mental well-being.^12^ Each construct is individually scored from 1 (no problems) to 5 (unable to do/extreme problems) and then an average score is calculated across the first three domains. It also includes a patient global assessment (PGA) which is scored 0–100. RAPID-3 consists of three measures: function, pain and PGA. Each is scored 0—10 and then summed for a total possible score from 0—30.^14^ FLARE-RA consists of 13 questions assessing a variety of symptoms. The total score is from 0 (no flare) to 10 (maximum flare).^17^ The RA-FQ consists of five questions assessing pain, function (physical activities and activities such as work, social or family life), fatigue and stiffness. Each construct is scored 0 (no difficulty) to 10 (extreme difficulty) and then summed for a total possible score from 0 to 50.^7^ Further details of each PRO are provided in online supplemental table S2.

### Statistical analysis

Two-sided Fisher’s exact or unpaired t-tests were used to determine any differences in baseline characteristics between the flare and remission groups. Summary scores across each of the PROs at each clinical visit were used in the analysis. To identify predictors of time-to-flare, univariable Cox regression models were built with time-varying covariates in which time-to-flare was the dependent variable and PRO scores were independent variables, excluding data from flare visits. In addition, we adjusted for age and sex as a priori confounders in multivariable models. Receiver-operating characteristic (ROC) curves were constructed to explore the utility of PRO increments for identifying flare. This involved using the largest adverse change in PRO from baseline to the point-of-flare or end of follow-up, with flare versus sustained remission as the outcome. Areas under the ROC curves (AUCs) were used to comparatively assess the discriminatory utility of each PRO (bootstrapping technique) with respect to flare, with PRO increment cut-points derived in each case by applying Youden’s index, enabling sensitivity, specificity, positive and negative predictive value (PPV, NPV) and likelihood ratios to be calculated. In post hoc stratified analyses, differences between AUCs (ΔAUC) were evaluated in SPSS.^18^ An exploratory analysis was conducted using locally estimated scatter smoothing (LOESS) regression method to visualise longitudinal changes in EQ5D Index, RAPID-3, FLARE-RA and RA-FQ scores in both groups prior to point-of-flare or end-of-study. K-Fold cross-validation was used to identify the best fitting model for each PRO.

8/58 (13.8%) flare and 19/63 (30.2%) remission participants had missing data for one or more clinical visits, largely due to the impact of COVID-19 on clinical visits. All participants were included in the analysis (with a piecewise constant assumption for Cox models) regardless of missing data, and no imputation was performed. All statistical analyses were conducted in the R computing environment (V.4.3.2, R Core Team) and IBM SPSS Statistics (V.29.0.1.1).

## Results

63 (52.1%) participants remained in DMARD-free remission and 58 (47.9%) had a flare during the study period (table 1). The median disease duration across all participants at baseline was 5.5 years. The median time to flare was 65 days (IQR 43–96). At baseline, the mean age of the cohort was 64.2 years. 74 (61.2%) were females and 113 (93.3%) were of white ethnic background. Flare events were more frequent in females (p=0.043). Median PRO scores were similar across remission and flare groups at baseline; the only exception was the median EQ5D PGA component which was marginally higher at baseline, indicating better self-assessed health status in patients who subsequently flared compared with those who did not (95 vs 90; p=0.015).

**Table 1:** Baseline characteristics

Characteristics	Overall population	Flare group	Remission group	P value*
Number of patients	121	58	63	
Time to flare (days)		65.0 (42.5–95.8)		
Disease duration (years)	5.5 (3.7–10.6)	5.5 (4.2–10.6)	5.1 (3.4–10.4)	0.155
Age (years), mean (SD)	64.2 (11.9)	64.8 (11.7)	63.5 (12.1)	0.543
Female, n (%)	74 (61.2)	41 (70.1)	33 (52.4)	0.043
Ethnicity, n (%)				0.602
White	113 (93.3)	55 (94.8)	58 (92.1)	
Asian	6 (5.0)	3 (5.2)	3 (4.8)	
Black	2 (1.7)	0	2 (3.2)	
Smoking status, n (%)				0.486
Current smoker	8 (6.6)	5 (8.6)	3 (4.8)	
Ex-smoker	64 (52.9)	32 (55.2)	32 (50.8)	
Never smoked	49 (40.5)	21 (36.2)	28 (44.4)	
EQ5D Index	0.8 (0.8–1.0)	0.8 (0.7–1.0)	0.8 (0.8–1.0)	0.242
EQ5D PGA	93.0 (85.0–98.0)	95.0 (90.0–98.1)	90.0 (80.0–97.5)	0.015
RA-FQ	3.0 (0–9.0)	2.0 (0–9.8)	3.0 (0–8.0)	0.811
FLARE-RA	0.8 (0–1.9)	0.8 (0–1.8)	0.5 (0–1.9)	0.803
HAQ-DI	0 (0–0.2)	0 (0–0.2)	0 (0–0)	0.165
MFI	62.0 (59.0–64.0)	62.0 (59.3–64.0)	62.0 (59.0–64.0)	0.689
RAPID-3	1.5 (0.5–3.3)	1.8 (0.8–4.2)	1.3 (0–3.0)	0.796

* p-P values for comparison between flare and non-flare group were obtained using Fisher’s exact test or unpaired t-test for dichotomous and continuous variables respectively.; p<0.05 was considered statistically significant. Data are median (IQR) unless otherwise specified. EQ5D: 5-level EuroQol-5D; RA-FQ: Flare Questionnaire; FLARE-RA: Flare assessment in ; HAQ-DI: Health Assessment Questionnaire Disability Index; MFI: ; RAPID-3: Routine Assessment of Index Data 3; PGA: .

EQ5DEuroQol-5 DimensionsFLARE-RAFactors that Limit sustAined Remission in rhEumatoid arthritisHAQ-DIHealth Assessment Questionnaire Disability IndexMFIMultidimensional Fatigue InventoryPGApatient global assessmentRA-FQRheumatoid Arthritis Flare QuestionnaireRAPID-3Routine Assessment of Patient Index Data 3

### Association between changes in PRO and flare

Univariate Cox regression confirmed changes in several PROs during follow-up were significantly associated with the hazard of flare (table 2). The strongest magnitude of association was seen for FLARE-RA, with a HR 1.29 (95% CI 1.12 to 1.48; p<0.001) for every 1-point increase in the score. Similar but less pronounced patterns were seen for RA-FQ (HR 1.04, 95% CI 1.061 to 1.07, p=0.01) and RAPID-3 (HR 1.12, 95% CI 1.06 to 1.18, p<0.001) scores. Consistent with these findings, the hazard of having a flare was associated with an analogous decrease (indicating reduced health status) in EQ5D Index (HR 0.03, 95% CI 0.00 to 0.33, p=0.005). These significant associations were maintained in multivariable analyses after adjusting for age and sex (table 2).

**Table 2:** Univariable and multivariable analyses of prediction of time to flare based on change from baseline patient-reported outcome scores

PRO	Unadjusted		Adjusted for age and sex	
	HR (95% CI)	P value	HR (95% CI)	P value
FLARE-RA	1.29 (1.12 to 1.48)	<0.001	1.29 (1.12 to 1.48)	<0.001
EQ5D Index	0.03 (0.00 to 0.33)	0.005	0.01 (0.00 to 0.17)	0.001
RAPID-3	1.12 (1.06 to 1.18)	<0.001	1.11 (1.05 to 1.17)	<0.001
RA-FQ	1.04 (1.01 to 1.07)	0.01	1.04 (1.01 to 1.07)	0.02

EQ5DEuroQol-5 DimensionsFLARE-RAFactors that Limit sustAined Remission in rhEumatoid arthritisPROpatient-reported outcomesRA-FQRheumatoid Arthritis Flare QuestionnaireRAPID-3Routine Assessment of Patient Index Data 3

### PROs for identification of flare

**Figure 1:**
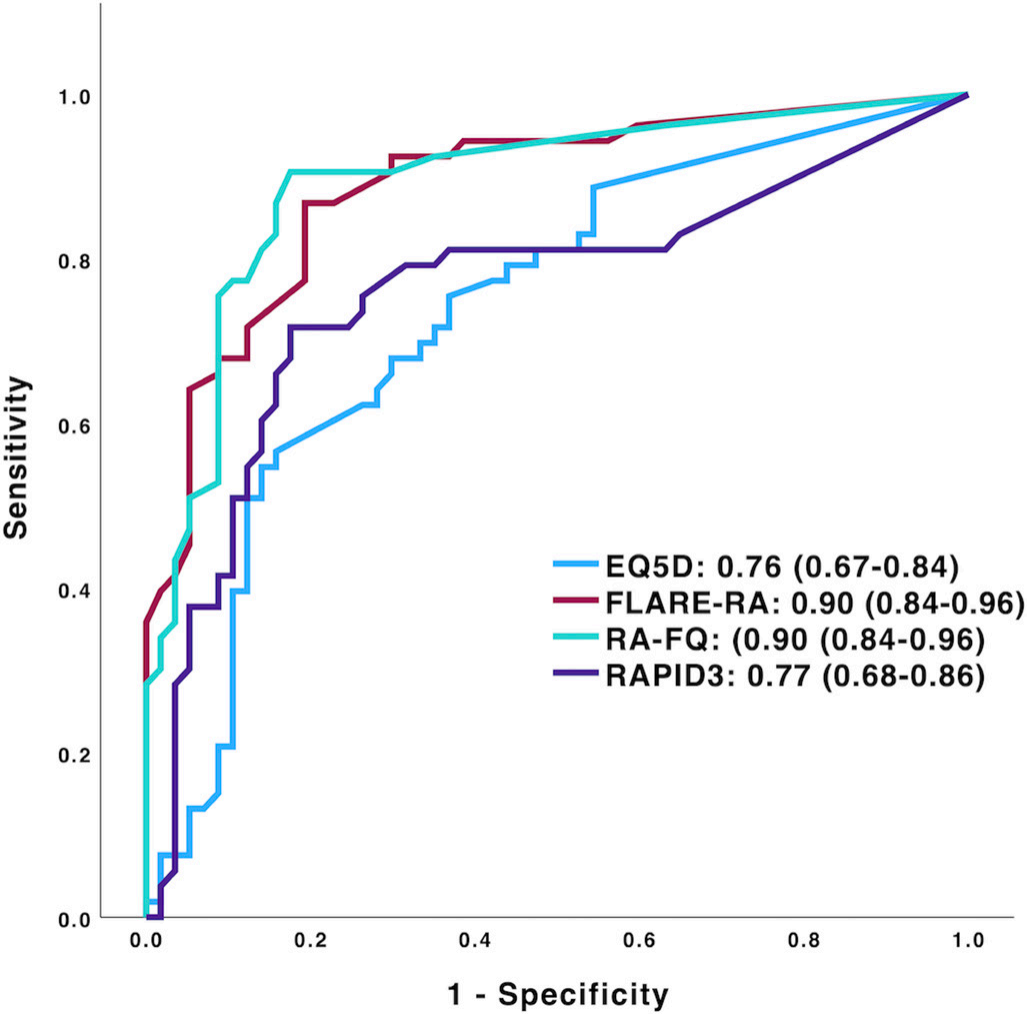
Receiver operating characteristic curves for change in patient-reported outcome scores from baseline to point-of-flare. Area under curve and 95% CI have been presented for each patient-reported outcome. EQ5D Index scores were inverted (negative values rendered positive) for purposes of plot generation only, to allow representation on the same axis. EQ5D, EuroQol-5 Dimensions; FLARE-RA, Factors that Limit sustAined Remission in rhEumatoid arthritis; RA-FQ, Rheumatoid Arthritis Flare Questionnaire; RAPID-3, Routine Assessment of Patient Index Data-3.

We then sought to prioritise a measure whose prospective monitoring from the point of DMARD cessation might optimally lead to prompt identification of flare during the 6-month follow-up period. ROC analysis showed that adverse changes in PRO scores from baseline were useful in detection of flare. Of those evaluated, RA-FQ and FLARE-RA were jointly optimal in this regard (both AUCs 0.90, 95% CI 0.84 to 0.96; p<0.001) (figure 1). Using the Youden index to objectively derive a cut-point that optimally balanced sensitivity with specificity, an increase in RA-FQ score of ≥5.5 would have identified 80% of incident or impending flares; by contrast, just 9% of individuals who did not record an increase of this magnitude flared during the same period (PPV 0.80, 95% CI 0.71 to 0.87; NPV 0.91, 95% CI 0.80 to 0.96). A FLARE-RA increase of >1.8 had similar utility. Analogous data is presented for all PROs in table 3. We further explored the potential of PRO monitoring to *predict* flare in this setting by excluding PRO values at time-of-flare. Although a modest discrimination was evident for some PROs using this approach, this was at best modest (eg, AUC 0.64, 95% CI 0.53 to 0.74 for RAPID-3; p=0.01) (online supplemental figure 1). Finally, we undertook *post hoc* stratified analyses, for example, comparing PROs’ utility among individuals whose disease duration was below the median for the cohort (5.5 years) with the remainder. Here, the discriminatory utility of RA-FQ for detecting flare was significantly greater for those with shorter disease duration (ΔAUC=0.13; nominal p=0.02; online supplemental figure 2), with similar trends apparent for other PROs. No such differences were seen for age.

**Table 3:** Minimal clinically important thresholds for PROs at point-of-flare

PRO	AUC (95% CI)	Youden’s Index	Proposed Cut-off	Sensitivity (95% CI)	Specificity (95% CI)	PPV (95% CI)	NPV (95% CI)	Positive LR (95% CI)	Negative LR (95% CI)
FLARE-RA	0.90 (0.84 to 0.96)	0.69	≥1.8	0.88 (0.76 to 0.95)	0.81 (0.70 to 0.90)	0.81 (0.71 to 0.88)	0.88 (0.79 to 0.94)	4.68 (2.78 to 7.86)	0.15 (0.07 to 0.31)
EQ5D Index	0.76 (0.67 to 0.84)	0.42	≥ −0.167	0.58 (0.44 to 0.71)	0.84 (0.73 to 0.92)	0.77 (0.64 to 0.86)	0.69 (0.62 to 0.76)	3.71 (2.01 to 6.83)	0.5 (0.36 to 069)
RAPID-3	0.77 (0.68 to 0.86)	0.52	≥2.75	0.68 (0.54 to 0.80)	0.84 (0.72 to 0.92)	0.79 (0.68 to 0.87)	0.74 (0.66 to 0.81)	4.14 (2.28 to 7.50)	0.38 (0.26 to 0.57)
RA-FQ	0.90 (0.84 to 0.96)	0.72	≥5.5	0.91 (0.80 to 0.97)	0.80 (0.68 to 0.89)	0.80 (0.71 to 0.87)	0.91 (0.80 to 0.96)	4.54 (2.72 to 7.58)	0.12 (0.05 to 0.27)

AUCarea under curveEQ5DEuroQol-5 DimensionsFLARE-RAFactors that Limit sustAined Remission in rhEumatoid arthritisLRlikelihood ratioNPVnegative predictive valuePPVpositive predictive valuePROspatient-reported outcomesRA-FQRheumatoid Arthritis Flare QuestionnaireRAPID-3Routine Assessment of Patient Index Data 3

### Longitudinal trends in PROs

**Figure 2:**
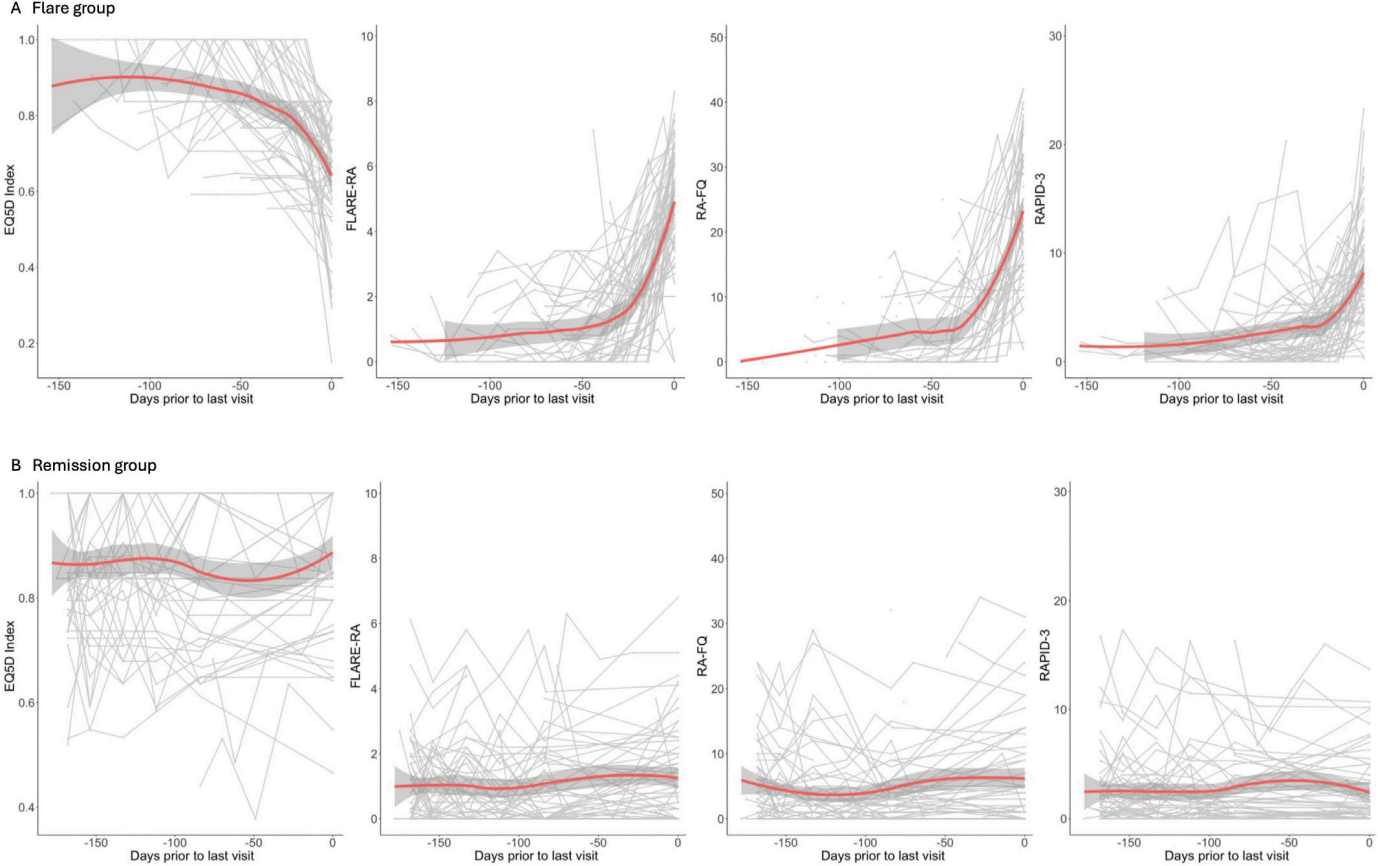
Longitudinal trends in patient-reported outcomes for flare (**A**) and remission (**B**) groups prior to the last clinical visit. Red lines show the fitted trends by loess regression, grey shading shows 95% CIs. The last clinical visit was defined as the point-of-flare for the flare group and the end of follow-up for the remission group. EQ5D, EuroQol-5 Dimensions; FLARE-RA, Factors that Limit sustAined Remission in rhEumatoid arthritis; RA-FQ, Rheumatoid Arthritis Flare Questionnaire; RAPID3, Routine Assessment of Patient Index Data-3.

An exploratory analysis using non-parametric LOESS plots showed longitudinal changes in PRO scores during the follow-up period. Figure 2 shows PRO scores in the period prior to flare or end-of-study time points among participants in flare and remission groups, respectively. We observed a clear decline in EQ5D Index (indicating declining health status) in the flare group prior to their flare, which accelerated as the flare time point was approached. The reciprocal pattern was seen for FLARE-RA, RAPID-3 and RA-FQ composites (indicating increasing arthritis symptoms). By contrast, participants who remained in DAS28-remission throughout the study had relatively unchanged scores for all these PROs.

## Discussion

This is the first prospective study to assess the value of longitudinal monitoring of PROs in prediction and detection of flare in a cohort of RA patients in drug-free remission. We found that the hazard of subsequent flare within 6 months following csDMARD discontinuation increases significantly for every increment in FLARE-RA, RA-FQ and RAPID-3 scores, or every EQ5D decrement, after correction for age and sex. We demonstrated that monitoring adverse changes in PROs from baseline over time has excellent ability to discriminate impending or incident flare from sustained remission during the first 6 months following csDMARD cessation. The approach therefore holds promise as a remote monitoring tool for timely identification and swift treatment of such events. Of the PROs assessed in this study, RA-FQ and FLARE-RA were jointly superior at identifying flare, with increments of ≥5.5 and ≥1.8 from baseline identifying at least 80% of flares, respectively. Furthermore, a *post hoc* stratified analysis revealed RA-FQ to be significantly more discriminatory in patients with shorter disease duration—perhaps reflecting a blunted perception of flare symptoms with the accrual of joint damage over time—and this finding warrants independent validation. However, our analysis did not confirm a clinically valuable predictive utility of PROs prior to point-of-flare, despite flare and remission Loess plots for each PRO suggesting earlier divergence. There are several plausible explanations for this observation. First, PRO scores observed over time among patients in stable remission (figure 2) display considerable background variability, such that only comparatively large changes that overcome this ‘noise’ will differentiate flare. Second, PROs are constructed using variables indicative of quality of life, functional status, disease activity, pain or flare itself, which have considerable overlap with components of DAS28 score. Third, due to the predefined timing of the follow-up visits and absence of remote monitoring between clinical visits, it is possible that we may have missed relevant information subsequent to the preflare visit but before the time of flare.

The potential of digital health applications in modern healthcare systems is increasingly recognised.^19^ Digital monitoring has various benefits in the context of chronic diseases including improved patient outcomes, reduced hospital visits^20^ and cost-saving benefits for both patients and healthcare providers.^21^ PROs have numerous benefits in this regard as they mitigate the need for physical examination or laboratory investigations and therefore can be rapidly completed both during routine outpatient clinic appointments and remotely through applications installed on smartphones and personal devices. More specifically, changes in symptoms can prompt patients to complete *ad hoc* PROs and thereby prompt clinical interventions via direct communication with healthcare providers. Deployment of public resources when patients need them most could thereby be facilitated. In the context of DMARD tapering, an increasing PRO score that reaches a predetermined threshold could trigger earlier clinical review and rapid re-escalation of treatment, or development of a flare plan, as required.

Our study adds to the growing body of evidence that supports the use of PROs for monitoring of RA patients. Previous studies examining the efficacy of telemedicine in RA patients have mostly been limited to patients with early RA or low disease activity.^22^ These have shown that remotely monitoring PROs can improve time to clinical remission,^23 24^ treatment adherence^23 25^ and function^23^ in people with early RA. Similarly, a French trial showed that, compared with conventional management, telemedicine can improve patient satisfaction and is associated with significant cost reductions in patients with RA initiating DMARDs.^26^ To our knowledge, no previous studies have examined PROs or remote monitoring in the context of DMARD withdrawal in RA. Our study is foundational to the design of future controlled trials to evaluate the effectiveness of such a strategy for preventing flares and delivering cost savings in this setting, without increased adverse outcomes. Only through such studies may the concept of ‘just-in-time’ adaptive intervention based on an individuals’ changing needs and context be realised.^27^ Critical to their conduct is the need to consider participant accessibility, including literacy, language and avoidance of digital exclusion.

Our findings should be considered in the context of certain limitations. First, the BIO-FLARE cohort consisted of older patients with a relatively long history of RA who were in remission on csDMARDs. Flare rates among individuals stopping biological DMARDs are known to differ, and our findings cannot necessarily be extrapolated to such settings. Second, serial measurements of HAQ-DI and MFI were not captured, precluding further consideration of these measures longitudinally. Third, some overlap exists between the individual constructs of the PROs used in our study (online supplemental table 2) and future research should aim to evaluate the effects of subcomponents of each PRO, to determine those with the most utility for predicting flares and help reduce the burden on patient reporting. Fourth, PRO measurements were limited to predefined clinical visit time points, whereas a remote monitoring system may have enabled measurement of changes between clinical visits and, therefore, may have led to better assessment of PRO trends and earlier identification of flare. Finally, we were only able to monitor flares that occurred within 24 weeks of csDMARD cessation, although this is when most flares will have occurred. An extended follow-up period is a priority for future investigation. It should also be noted that the clinical definition of remission employed in BIO-FLARE (DAS28-CRP<2.4), while generally accepted, is not a gold standard in itself, comprising as it does subjective elements. However, validated definitions for remission using more ‘objective’ outcome measures are yet to be developed.^28^

In summary, we have shown that monitoring changes in subjective elements of RA disease activity captured by PROs may predict and identify flare promptly in people who choose to stop csDMARDs. Our findings support the potential value of remote PRO monitoring for this purpose, informing the design of controlled trials to formally evaluate such a strategy.

## supplementary material

10.1136/rmdopen-2025-005442 online supplemental file 1

## Data Availability

Data are available on reasonable request.
