# Supplementary material for: Patient-reported outcomes as early warning signs of flare following drug cessation in rheumatoid arthritis
Source: RMD Open. 2025 Apr 1;11(2):e005442. doi: 10.1136/rmdopen-2025-005442 (PMC11962807; doi:10.1136/rmdopen-2025-005442)

**APPENDIX**

Table of Contents

[Table S1. Patient reported outcome measures collected during the study 2](#_Toc190953029)

[Table S2. Components assessed in each patient reported outcome measure 3](#_Toc190953030)

[Figure S1. Receiver operating characteristic curves for change in patient reported outcome scores from baseline to pre-flare visit. 4](#_Toc190953031)

[Figure S2. Receiver operating characteristic curves for change in patient reported outcome scores from baseline to flare visit based on duration of RA. 5](#_Toc190953032)

# Table S1. Patient reported outcome measures collected during the study

| **Tool** | **Time points collected (weeks)** | **Scoring** |
| --- | --- | --- |
| HAQ-DI[11] | 0, 24 | 3-point scale, overall score is an average across all domains.  Overall score: 0 (no difficulty) to 3 (unable to do) |
| EQ5D Index[12] | 0, 2, 5, 8, 12, 24 | 5-point scale, 1 (no problem) to 5 (extreme problem), overall score is an average across all domains.  Overall score: 0 (worst health) to 1 (best health). |
| EQ5D PGA[12] | 0, 2, 5, 8, 12, 24 | 1 to 100 with 100 indicating the best overall health status |
| RAPID-3[14] | 0, 2, 5, 8, 12, 24 | Separated into function (out of 10), pain (out of 10) and global status (out of 10).  Overall score: 0 to 30 with higher scores indicating greater disease activity. |
| FLARE-RA[17] | 0, 2, 5, 8, 12, 24 | 13 questions rated on a 6-point scale (1=absolutely true; 6=completely untrue).  Overall score: 0 (no flare) to 10 (maximum flare). |
| RA-FQ[7] | 0, 2, 5, 8, 12, 24 | 5 questions rated 0 (no difficulty) to 10 (extreme difficulty).  Overall score: 0 to 50 with 50 indicating extreme difficulty. |
| MFI[13] | 0, 24 | 20 questions rated on 5-point scale (1 = yes that is true; 5 = no that is not true).  Overall score 20 to 100 with 100 indicating higher levels of fatigue. |

EQ5D: 5-level EuroQol-5D; RA-FQ: Rheumatoid Arthritis Flare Questionnaire; FLARE-RA: Flare assessment in rheumatoid arthritis; HAQ-DI: Health Assessment Questionnaire Disability Index; MFI: Multidimensional Fatigue Inventory; RAPID-3: Routine Assessment of Patient Index Data 3; PGA: Patient global assessment.

# Table S2. Components assessed in each patient reported outcome measure

| **Tool** | **Pain** | **Function** | **Fatigue** | **Stiffness** | **Swelling** | **Mood** | **Sleep** | **Overall health** |
| --- | --- | --- | --- | --- | --- | --- | --- | --- |
| EQ5D | ✓ | ✓ | x | x | x | ✓ | x | ✓ |
| RA-FQ | ✓ | ✓ | ✓ | ✓ | x | x | x | x |
| FLARE-RA | ✓ | ✓ | ✓ | ✓ | ✓ | ✓ | ✓ | x |
| RAPID-3 | ✓ | ✓ | x | x | x | ✓ | ✓ | ✓ |
| MFI | x | x | ✓ | x | x | ✓ | x | x |
| HAQ-DI | ✓ | ✓ | x | x | x | x | x | ✓ |

# Figure S1. Receiver operating characteristic curves for change in patient reported outcome scores from baseline to pre-flare visit.

Area under curve (AUC) and 95% CI have been presented for each patient reported outcome. EQ5D scores were inverted (negative values rendered positive) for purposes of plot generation only, to allow representation on the same axis.


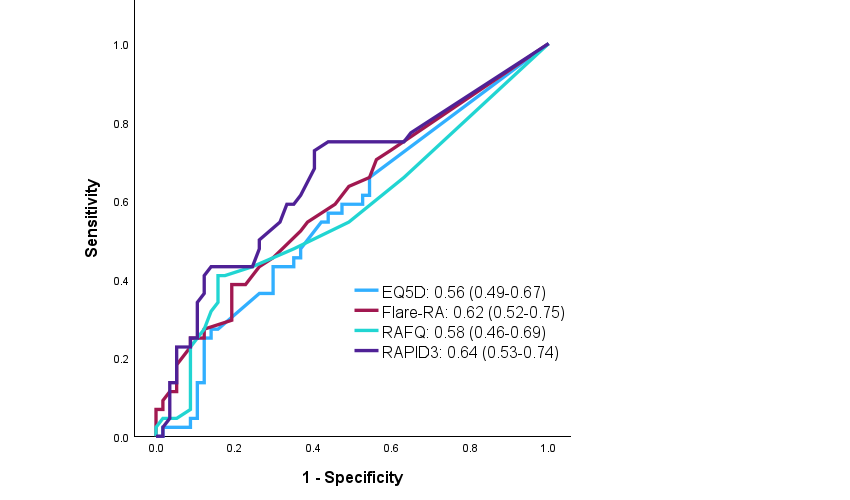


# Figure S2. Receiver operating characteristic curves for change in patient reported outcome scores from baseline to flare visit based on duration of RA.

Area under curve (AUC) and 95% CI have been presented for each patient reported outcome. EQ5D scores were inverted (negative values rendered positive) for purposes of plot generation only, to allow representation on the same axis. ΔAUC represents the difference in AUC between short (<5.5 years) and long (≥5.5 years) disease duration.


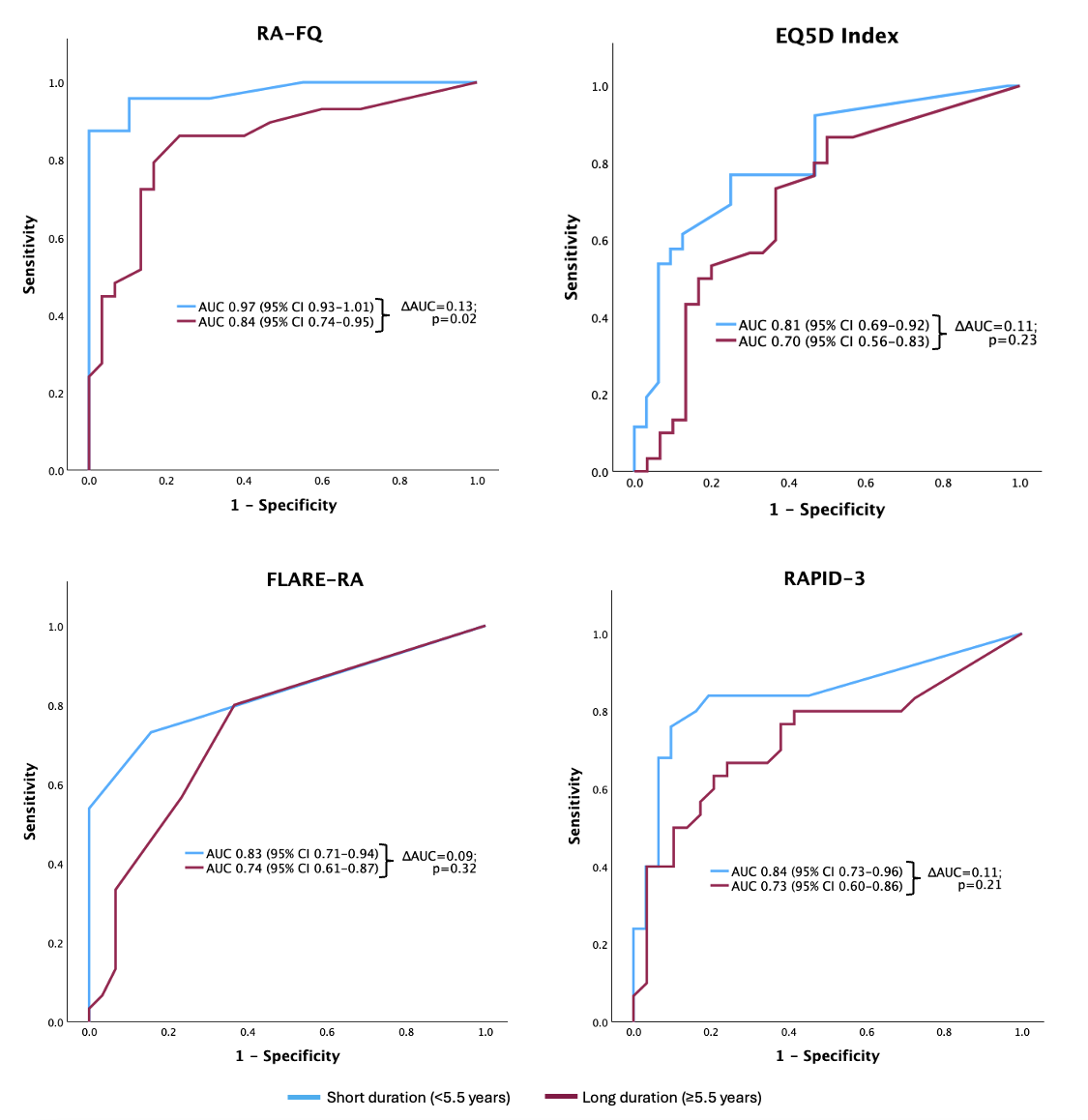

Supplement: online supplemental file 1 [file rmdopen-11-2-s001.docx]
